# Intensive virtual reality and robotic based upper limb training compared to usual care, and associated cortical reorganization, in the acute and early sub-acute periods post-stroke: a feasibility study

**DOI:** 10.1186/s12984-019-0563-3

**Published:** 2019-07-17

**Authors:** Jigna Patel, Gerard Fluet, Qinyin Qiu, Mathew Yarossi, Alma Merians, Eugene Tunik, Sergei Adamovich

**Affiliations:** 1 Department of Rehabilitation and Movement Sciences, School of Health Professions, Rutgers University, The State University of New Jersey, 65 Bergen Street, Newark, NJ 07107 USA; 2 Movement Neuroscience Laboratory, Department of Physical Therapy, Bouve College of Health Sciences, Movement and Rehabilitation Science, Northeastern University, 308C Robinson Hall – 360 Huntington Avenue, Boston, MA 02115 USA; 3 Department of Biomedical Engineering, New Jersey Institute of Technology, 616 Fenster Hall – 323 Dr. MLK Jr. BLVD, Newark, NJ 07102 USA

**Keywords:** Stroke, Upper limb, Acute, Early sub-acute, Virtual reality, Robotic therapy, Transcranial magnetic stimulation

## Abstract

**Background:**

There is conflict regarding the benefits of greater amounts of intensive upper limb rehabilitation in the early period post-stroke. This study was conducted to test the feasibility of providing intensive therapy during the early period post-stroke and to develop a randomized control trial that is currently in process. Specifically, the study investigated whether an additional 8 h of specialized, intensive (200–300 separate hand or arm movements per hour) virtual reality (VR)/robotic based upper limb training introduced within 1-month post-stroke resulted in greater improvement in impairment and behavior, and distinct changes in cortical reorganization measured via Transcranial Magnetic Stimulation (TMS), compared to that of a control group.

**Methods:**

Seven subjects received 8–1 h sessions of upper limb VR/robotic training in addition to their inpatient therapy (PT, OT, ST). Six subjects only received their inpatient therapy. All were tested on measures of impairment [Upper Extremity Fugl-Meyer Assessment (UEFMA), Wrist AROM, Maximum Pinch Force], behavior [Wolf Motor Function Test (WMFT)], and also received TMS mapping until 6 months post training. ANOVAs were conducted to measure differences between groups across time for all outcome measures. Associations between changes in ipsilesional cortical maps during the early period of enhanced neuroplasticity and long-term changes in upper limb impairment and behavior measures were evaluated.

**Results:**

The VR/robotic group made significantly greater improvements on UEFMA and Wrist AROM scores compared to the usual care group. There was also less variability in the association between changes in the First Dorsal Interosseus (FDI) muscle map area and WMFT and Maximum Force change scores for the VR/robotic group.

**Conclusions:**

An additional 8 h of intensive VR/robotic based upper limb training initiated within the first month post-stroke may promote greater gains in impairment compared to usual care alone. Importantly, the data presented demonstrated the feasibility of conducting this intervention and multiple outcome measures (impairment, behavioral, neurophysiological) in the early period post-stroke.

## Background

Approximately 795,000 new or recurrent strokes occur each year in the United States and the prevalence of chronic stroke is approximately seven million [1]. It is a leading cause of adult long-term disability in the United States with the financial burden of related care among the fastest-growing expenses for Medicare [1]. Proportionally more stroke survivors are left with upper extremity impairment and disability than that of the lower extremity [2]. At 6 months post-stroke only 5–20% achieve full return of arm function [3, 4]. It is thus imperative to develop and test innovative upper extremity training protocols that are based on sound principles of motor learning, and also to compare changes in impairments, behavior, and brain organization to help identify the neural substrates of recovery.

There is a time-limited period of unique neuroplasticity post ischemic stroke that lasts about one to 3 months in humans. This plasticity mediates spontaneous biological recovery and produces enhanced responsiveness to rehabilitative interventions introduced during that time [5]. It is believed that during this time of unique plasticity, impairment based recovery is maximal and is mediated from both of these related processes - spontaneous recovery and enhanced responsiveness to training [5]. Consequently, it would be logical to assume that additional hours of intensive training initiated within the acute and early sub-acute period post-stroke (acute: 1–7 days post, early sub-acute: second week - 3 months post [6]) would interact with this distinct type of plasticity and result in better outcomes compared to conventional rehabilitative care. Careful review of the literature suggests that the relationship may not be so straightforward. For example, a 2014 meta-analysis found a positive relationship between increased therapy time and clinical measures of function and impairment overall [7]. However, other individual studies (including a large randomized controlled trial (RCT)), and a sub-analysis from a 2004 review, that have focused on therapy within this early phase, and specifically compared higher amounts of upper limb therapy to lower amounts, found no statistically significant benefit of higher amounts of intervention on different outcomes measured at varied time points post training [8–12]. Additionally, an influential study by Dromerick et al. found that 3 h of Constraint Induced Movement Therapy (CIMT) led to worse outcomes on the Action Research Arm Test (ARAT) - [13] when compared to 2 h of CIMT or 2 h of conventional occupational therapy [14].

Mechanisms of neuroplasticity such as the formation of new synaptic connections with concomitant modification in the cortical excitability and somatotopic remapping can be positively influenced by training methods that are developed from established principles of motor learning [15–17]. The study presented here was performed to determine feasibility, and to help develop a large scale randomized controlled trial (RCT) that we are currently conducting at a nationally recognized rehabilitation center [(https://ClinicalTrials.gov (NCT03569059)]. Specifically, the research was formulated to help fill a gap in the literature by testing whether gains in upper limb impairment and behavior are greater if an additional 8 h of intensive, motor learning based VR/robotic training (VR group) is provided during the first month post-stroke compared to usual care alone (UC group). The VR/robotic system enables 200–300 activity based hand and arm movements per hour of training. This volume is necessary to elicit neuroplastic changes [18], and is much greater than the average of 40.64 (32.14) repetitions per session provided by conventional rehabilitation in similar settings [19]. Bilateral cortical reorganization was evaluated via changes in Transcranial Magnetic Stimulation (TMS) induced maps.

In contrast to trends in the literature, we hypothesized that participants in the VR/robotic training group would demonstrate greater gains on both impairment (assessed with the Upper Extremity Fugl-Meyer Assessment - UEFMA [20], wrist active range of motion – Wrist AROM, and Maximum Pinch Force) and behavioral measures (assessed with the Wolf Motor Function Test - WMFT [21]) compared to the UC group due to preferential effects of the VR/robotic training on the unique plasticity occurring during the first month post-stroke.

Topographic patterns of reorganization of the corticospinal system can be quantified using TMS induced motor evoked potentials (MEPs) to assay the integrity of the sensorimotor cortex representation of arm and hand muscles. Although some studies using TMS mapping to track ipsilesional motor reorganization over the first months to 1 year following stroke have indicated that increased excitable areas in the ipsilesional hemisphere are associated with recovery of the upper limb [22–25], other studies have found no change in ipsilesional excitable area over the same period [26, 27]. This contradiction of findings is part of a larger current controversy over the interpretation of M1 reorganization as it relates to recovery. Further research is necessary to better understand the complex relationship between effector specific M1 reorganization, amenability of the effector to training, and behavioral and impairment based gains. To date, we know of only two studies that have sought to quantify the neuroplastic changes (via TMS mapping) evoked by an intervention in this same early stage following stroke [28, 29]. Results from Boake et al. (2007) indicated that an increased number of MEP-active sites in the ipsilesional hemisphere was associated with increased functional improvement in individuals receiving CIMT compared to controls receiving usual care. In contrast, Platz et al. (2005) did not find any change in the number of active sites in their two treatment groups (Bobath or Impairment Oriented Arm Training). We surmised that if greater impairment and behavioral-based gains in the VR group is attributed to bolstering neuroplastic changes that normally occur during this time period, the VR/robotic training would be associated with a greater expansion in ipsilesional M1 hand muscle representations (measured via TMS) compared to the hand muscle territory measured in the UC group (which would be reflective of neuroplastic changes attributed to spontaneous mechanisms and usual care).

## Methods

### Subjects and protocol

Thirteen subjects were recruited from a small (20 bed) inpatient rehabilitation unit of a suburban hospital and participated in this feasibility study following institutionally approved informed consent. After initial screening by the department’s physician, a physical therapist screened subjects based on the following criteria: Inclusion: 1) within 1 month after first time unilateral ischemic or hemorrhagic stroke, 2) between the ages of 30 and 80, 3) participants were able to actively: perform mass finger flexion and extension a minimum of 5 degrees, 5 times in 1 min with their arm at the side of their body and their elbow flexed; perform elbow extension a minimum of 5 degrees, 5 times in 1 min (returning to original position after each movement); lift the affected hand up off of their lap and place it onto a table located in front of them (table height a few inches taller than lap), and 4) participants were able to tolerate passive ROM of the shoulder to 90 degrees in flexion and abduction without neck, shoulder or hand pain. Exclusion: 1) severe spasticity (Modified Ashworth score of 3 or greater [30]), 2) cognitive deficits rendering them unable to follow three step commands or attend to a task for at least 10 min (based on review of the Speech Therapist’s evaluation using the Montreal Cognitive Assessment [31]), 3) hemispatial neglect rendering them unable to interact with an entire twenty-four inch computer screen (based on review of the physiatrist’s admission evaluation), 4) proprioceptive loss that rendered them unable to interact with a virtual environment without looking at their hands (tested clinically by the physical therapist), and 5) unstable blood pressure and oxygen saturation responses to activity. Exclusion criteria for TMS included: 1) diagnosis of epilepsy, 2) implanted metal in the head or neck, 3) the subject was pregnant, and 4) implanted electronic devices. After screening and consent, participants were alternately assigned to the treatment (VR) group or usual care (UC) group.

Virtual reality protocol (VR group): This group began training as inpatients within the first month post-stroke. This was initiated as soon as possible after PRE testing was completed. The VR group received eight 1-h sessions (1 h of training provided 200–300 separate hand or arm movements) of hand-focused upper extremity VR/robotic training in addition to their usual 3 h of rehabilitation (physical, occupational, and speech Therapy - on consecutive days Monday-Friday).

Usual care protocol (UC group): This group of participants were also inpatients within the first month post-stroke and received a combination of physical, occupational, and speech therapy for 3 h a day. This therapy consisted of adaptive and progressive task and impairment based therapy including strengthening, ROM, mobility, activities of daily living, and transfer training. Subjects with finger and wrist weakness typically also received electrical stimulation of the finger and wrist extensor muscles.

### VR/robotic system

For the intensive VR/robotic training, we used the NJIT-RAVR system. This system provides an adaptive and progressive motor learning environment through sensory and perceptual modifications such as force modulation, activity and workplace scaling, gain manipulation and error augmentation [32]. Notably, the NJIT-RAVR system was shown to be effective at reducing impairments in a chronic stroke population [33–35].

#### Hardware

The NJIT-RAVR system comprises both an arm training robot (Haptic Master [Moog NCS, The Netherlands]) and an integrated system for the hand consisting of an instrumented measurement glove (CyberGlove [Immersion, USA]), a cable actuated hand exoskeleton that facilitates finger extension for those persons with more severe impairment (CyberGrasp [Immersion, USA]), and a 3-dimensional magnetic tracking system that tracks hand and arm position (TrackSTAR™ [Ascension Technology, USA]) – the NJIT Track–Glove System. The Haptic MASTER is an admittance-controlled robot with six degrees of freedom. A three-dimensional force sensor measures the external force exerted by the user on the robot. In addition, it provides tracking of multiplanar movements in a 3D workspace and enables programmable haptic effects, such as variable anti-gravity support, springs and dampers, and haptic objects, such as walls, floors, tables and other complex-shaped objects [33, 35]. The users interface with the Haptic Master via a forearm trough that extends through the gimbal, allowing for partial support of the weight of the arm as needed, while maintaining the ability to produce pronation and supination movements. It was individually programmed to provide assistance to lower functioning subjects with progressive adaptations that lessened the help provided as subjects improved over time.

#### Training simulations and interventions

The VR environment was developed with the Virtools 4.0 software package (Dassault Systemes, Velizy-Villacoublay, France) and a VRPack Plug-in that communicates with an open source Virtual Reality Peripheral Network (VRPN) interface. The NJIT-RAVR robotic system that interfaces with our suite of impairment and activity based VR simulations was used to train the hand and arm separately. This training system can be readily adapted in terms of speed, accuracy, amount of assistance provided by the robots, and the ratio of patient movement amplitude to avatar movement amplitude. The treatment group performed three simulations for the hand, and three for the arm - training approximately 10 min on each of the six simulations during each session. Each training simulation was designed to use an activity to address an impairment commonly experienced by persons with stroke. The hand simulations consisted of the games: Monkey Business, Space Pong, and Piano Trainer. Their forearm was supported on a table during these hand activities. The arm simulations consisted of the games: Space Ship, Hammer Trainer, and Placing Cups (please refer to Fluet et al. 2017 for details [36]). The CyberGrasp was used initially with persons with severe hand impairment who could not extend their fingers without assistance [37, 38].

### Outcome measures

All outcomes were measured at baseline (PRE), immediately post intervention (POST), and again one (1M) and 6 months (6M) after the intervention.

#### Impairment (body structure/function) measures

1. The Upper Extremity Fugl-Meyer Assessment (UEFMA): is an index of global UE motor recovery at an impairment level. The arm subsection was used with a total score of 66. This test measures single and multi-joint movement in and out of synergy, digit individuation, speed, dysmetria, ataxia, and reflexes. This is a widely used tool that is both reliable and valid in acute stroke populations [20, 39–41].
2. Wrist active range of motion (Wrist AROM): measures the average difference between maximum active wrist flexion and extension. This was measured using an industry standard, precise 3-dimensional magnetic tracking system that tracks hand and arm position (TrackSTAR™ [Ascension Technology, USA] – precision: 1.4 mm RMS, 0.5 degrees RMS). To increase reliability of the measure, the same person followed the same, set protocol at each test session [36, 38, 42].
3. Maximum pinch force: measures the maximum voluntary force that a subject can exert on an industry standard, precise force sensor [ATI Nano17™ force sensor (ATI Industrial Automation, USA) – precision: 0.318 g-force] held between their paretic thumb and index finger. Larger numbers indicate stronger pinch force. Subjects were given two attempts and the largest pinch force value was used**.** To increase reliability of the measure, the same person followed the same, set protocol at each test session [36, 38, 42].

#### Behavioral measure

The Wolf Motor Function Test (WMFT): measures participants’ capacity to use their recovering motor abilities to perform goal-oriented tasks. It is a quantitative measure of upper limb motor ability assessed via timed functional tasks. It is reliable and valid for use in the stroke population [21]. The log of the mean timed scores for 15 items was used in this study (weight to box and grip strength were not measured).

#### TMS mapping procedure (previously described in Yarossi et al. 2014 [43])

Surface electromyographic activity (EMG, Delsys Trigno, at 2 kHz) was recorded to measure the MEPs elicited by TMS. EMG was recorded from 5 hand muscles contralateral to the stimulation side: first dorsal interosseus [FDI], abductor pollis brevis [APB], abductor digiti minimi [ADM], flexor digitorum superficialis [FDS], and the extensor digitorum communis [EDC]. Motion of the contralateral arm was limited during TMS mapping by securing the arm and hand in a splint and via verbal cueing. To ensure spatial TMS precision for the repeated assessments, each subject’s head was coregistered to a canonical high-resolution anatomical MRI for frameless neuronavigation (Advanced Neuro Technology). All TMS measures were taken at rest and background EMG was monitored to ensure that muscles remained relaxed. The TMS coil (Magstim, 70 mm double coil) was held tangential to the scalp, with the handle held posteriorly and at 45° off the sagittal plane [44]. MEPs were sampled until the location with the largest MEP was determined [45, 46]. This method affords high intra- and inter-experimenter reliability [46], has been cross-validated with fMRI, and is robust in identifying the location of greatest activation for a given muscle [47]. Resting motor threshold (RMT) was determined at this location as the minimum intensity required to elicit MEPs > 50 uV in the FDI muscle on 50% of 6 sequential trials [48]. The hotspot and RMT were determined at each mapping session. All mapping was performed with the stimulation intensity set to 110% of the determined RMT [49]. A 7x7cm area surrounding the motor hotspot was marked using the neuronavigation software to provide consistent map boundaries. One hundred and fifty TMS pulses were delivered at a 4 s interstimulus interval within the grid boundaries with special attention paid to regions surrounding the hotspot territory. Real time feedback of multi-muscle MEPs and neuronavigated coil position was used to maximize the map information obtained by increasing the density of points in excitable and the ‘hotspot’ region while giving less attention in far-away non-responsive areas [50]. Mapping procedures were conducted for both the ipsilesional and contralesional hemispheres. The MEP for each stimulation point was calculated as the peak-to-peak amplitude of the EMG signal 20-50 ms after the TMS pulse.

#### TMS mapping analysis

Map area has been used extensively to describe sensorimotor cortex reorganization after stroke [51]. A threshold of 50uV was used to identify MEPs from background EMG [49]. MEP amplitudes and stimulation points were interpolated to a 7 × 7 cm mesh of 0.375 mm resolution (centered on the M1 hotspot) using cubic surface interpolation [52, 53] allowing comparisons across maps and sessions. Extent of the representation producing corticospinal output (MEPs) for individual muscles, or map area, was calculated using double trapezoidal integration of the interpolated map [43].

### Statistical analysis

Baseline status between groups was compared using Mann-Whitney U tests. A 2-way mixed ANOVA was conducted with a between factor of treatment Group (VR and UC) and a within factor of Time (PRE, POST, 1M, 6M) to evaluate the difference over time on impairment and behavioral measures. Effect size using Partial Eta Squared (η^2^) is provided for all findings to show the amount of variance in the outcome variables explained by group membership. This was used in part to determine the sample sizes required for the RCT. Log WMFT and Wrist AROM data were normalized prior to performing the ANOVAs due to issues with normality in these data sets. The other two outcomes had no such issues (UEFMA PRE and Wrist AROM PRE: SW(13) = 0.944, *p* = 0.513 and SW(11)) = 0.923, *p* = 0.36 respectively). PRE to 6 M changes in ability to perform items on the WMFT were evaluated using a Mann-Whitney U test. Alpha was set at 0.05 for all comparisons. The association between changes in ipsilesional FDI muscle area representations during the early, critical period of enhanced neuroplasticity and long-term Maximum Pinch Force and WMFT change scores was evaluated via scatterplots.

## Results

Thirteen individuals with first time stroke occurring less than 1 month prior to enrollment participated in the study. There were no statistically significant differences in age, days post-stroke, or in UEFMA scores between groups at baseline – PRE (Mann-Whitney U test – age: U = 18, *p* = 0.67, days post-stroke: U = 19.5, *p* = 0.825, UEFMA: U = 19.5, *p* = 0.83). Participant characteristics are listed in Table 1. All training was well tolerated without adverse incidents such as fatigue, medical complications, or interference with regularly scheduled therapies.

**Table 1:** Participant characteristics

Group/Subject	Age	Sex	Days post-stroke	Lesion location	UEFMA at PRE
VR1	45	M	12	Corona radiata and putamen	32
VR2	62	F	8	R midle cerebral artery	47
VR3	76	M	7	L frontal, parietal and occipital cortex	33
VR4	43	F	7	R middle and anterior cerebral arteries	31
VR5	60	M	7	L periventricular white matter	11
VR6	53	M	13	R temporal and parietal cortex	21
VR7	60	M	30	R posterior limb of the internal capsule and putamen	24
Mean (SD)	57.14(11.3) years		12(8.3) days		28.43(11.3)
UC1	59	F	6	L internal capsule	49
UC2	80	F	11	R frontal and insular cortex	19
UC3	52	M	7	L pons	30
UC4	70	M	7	L basal ganglia	21
UC5	56	M	17	L thalamus	49
UC6	55	M	30	R deep matter under the frontal lobe and R basal ganglia and thalamus	4
Mean (SD)	62(10.8) years		13(9.3) days		28.67(17.8)

### Impairment and behavioral outcomes

**Fig. 1:**
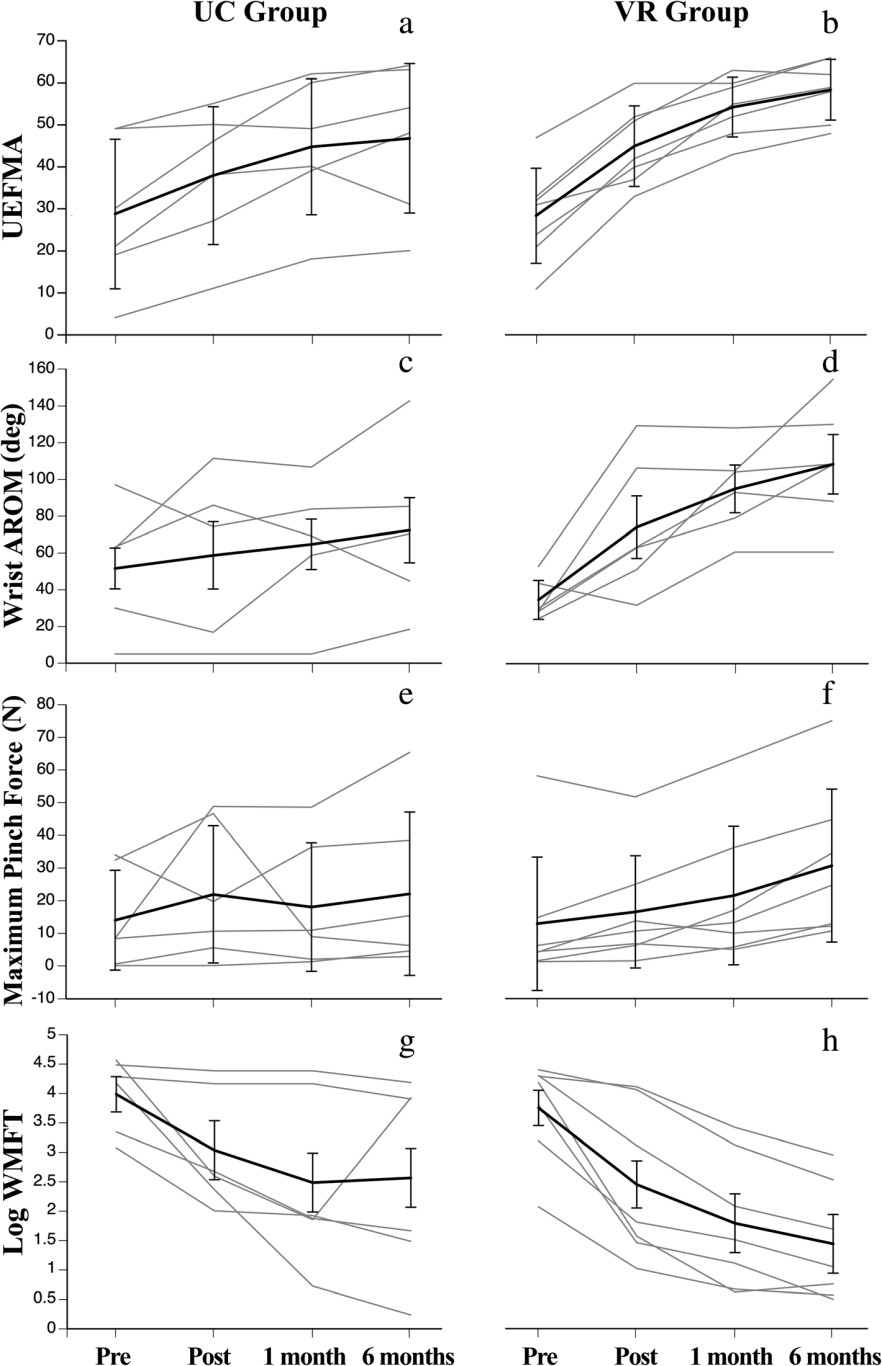
Individual data for UC (left) and VR (right) groups for all measures. UEFMA: **a** and **b**, Wrist AROM: **c** and **d**, Max Pinch Force: **e** and **f**, Log WMFT: **g** and **h**

Mann-Whitney U tests revealed no significant differences between groups at PRE for all four measures, indicating baseline function was similar between the two groups. Table 2 shows the results of these tests at PRE, as well as the means (standard deviations) for all outcome measures, for both groups at each time level (non-normalized values). A 2-way mixed ANOVA with a within factor of Time and a between factor of Group was used to test for main effects and interactions for the four impairment and behavioral outcomes (Table 3). Log WMFT and Wrist AROM data were normalized prior to performing the ANOVAs due to issues with normality in these two data sets. Effect size using Partial Eta Squared (η^2^) is provided for all findings. A Time X Group interaction was significant for the UEFMA [F(3,33) = 3.59, *p* = 0.024, η^2^ = 0.246] and Wrist AROM [F(3,27) = 3.93, *p* = 0.019, η2 = 0.304]. Preplanned contrasts (Tukey’s Least Significant Difference) between the two groups to test for differences in the amount of change from PRE to 6M are provided for the significant interactions. For the UEFMA, between group differences in PRE to 6M change scores were significant and greater for the VR group [F(1,11) = 5.83, *p* = 0.034, η^2^ = 0.346]. For Wrist AROM, between group differences in PRE to 6M change scores were significant and also greater for the VR group [F(1,9) = 5.342, *p* = 0.046, η^2^ = 0.372]. Importantly, 6/7 VR subjects versus only 2/6 UC subjects surpassed the minimal clinically important difference (MCID – value of 9 or 10) for the UEFMA from PRE to POST (during the training period) [54]. A Time X Group interaction was not significant for Log WMFT [F(3,33) = 1.18, *p* = 0.332, η^2^ = 0.097] and Maximum Pinch Force [F(1.81,19.96) = 1.02, *p* = 0.372, η2 = 0.085] scores. As well, the main effects of Group and Time were not significant for the WMFT and Maximum Pinch Force. PRE to 6M changes scores for the number of WMFT items performed were not significantly greater in the VR group [5.57(3.4)] than in the UC group [3.12(3.1)]; Mann-Whitney U test: U = 12.5, *p* = 0.22]. Figure 1 shows the individual data over time for all four measures.

**Table 2:** Mann-Whitney U test results at PRE and means (SDs) for all measures over time

Outcome/Group	PRE/between group Mann-Whitney U tests	POST	1M	6M
UEFMA				
VR	28.43(11.3)	45(9.6)	54.29(7.1)	58.43(7.2)
UC	28.67(17.8)	37.83(16.4)	44.67(16.2)	46.67(17.8)
	U = 19.5, *p* = 0.83			
Wrist AROM (deg)				
VR	34.75(10.6)	74.27(17)	95.11(13)	108.51(16.2)
UC	51.62(11.1)	58.72(18.3)	64.71(13.7)	72.36(17.7)
	U = 9, *p* = 0.27			
Max Pinch Force (N)				
VR	12.89(20.4)	16.49(17.2)	21.47(21.2)	30.65(23.4)
UC	14.01(15.3)	21.91(21)	18.05(19.7)	22.09(25)
	U = 20, *p* = 0.89			
Log WMFT (sec)				
VR	3.75(0.3)	2.45(0.4)	1.79(0.5)	1.44(0.5)
UC	3.99(0.3)	3.04(0.5)	2.49(0.5)	2.57(0.5)
	U = 20.5, *p* = 0.94

**Table 3:** Two-Way Mixed ANOVA results for all measures

Test	Time	Group	TIME X Group
UEFMA	F(3,33) = 60.44	F(1,11) = 1.02	F(3,33) = 3.59
	*p* < 0.001	*p* = 0.333	*p* = 0.024
Wrist AROM (deg)	F(3,27) = 11.58	F(1,9) = 0.80	F(3,27) = 3.93
	*p* < 0.001	*p* = 0.394	*p* = 0.019
Maximum Pinch Force (N)	F(1.81,19.96) = 0.006	F(1,11) = 0.005	F(1.81,19.96) = 1.02
	*p* = 0.99^*^	*p* = 0.944	*p* = 0.372^*^
Log WMFT (sec)	F(3,33) = 0.094	F(1,11) = 0.731	F(3,33) = 1.18
	*p* = 0.963	*p* = 0.411	*p* = 0.332

^*^Greenhouse-Geisser corrected

### TMS maps of cortical representation

**Fig. 2:**
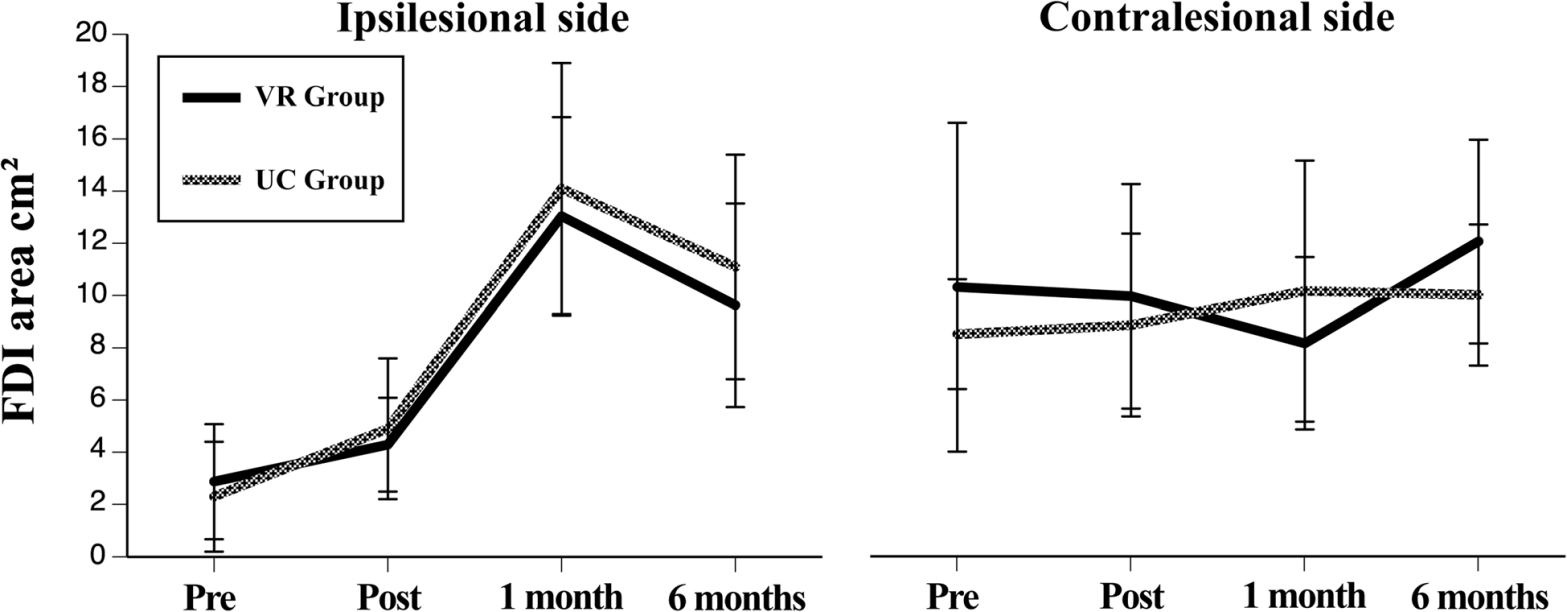
Comparison of ipsilesional and contralesional TMS maps for the FDI muscle

Six individuals in the VR and 5 in the UC group met the inclusion criteria for TMS mapping. TMS maps of the cortical representation of five hand muscles (FDI, APB, ADM, FDS, EDC) were obtained bilaterally in these individuals. The maps representing the FDI muscle are presented here (Fig. 2). The ispilesional cortical area representing the FDI muscle in both treatment groups was reduced compared to the contralesional side at PRE. The ipsilesional TMS map area for the FDI muscle increased from PRE to POST and POST to 1M(significant for both study groups, at *p* < 0.05 for PRE to 1M) with a non-significant reduction in size from 1M to 6M for both groups. There was no difference between groups over time ipsilesionally. Contralesional area for the FDI muscle monotonically increased from PRE to 6M in the UC group. Conversely, in the VR group, contralesional area decreased from PRE to 1M and then increased from 1M to 6M.

**Fig. 3:**
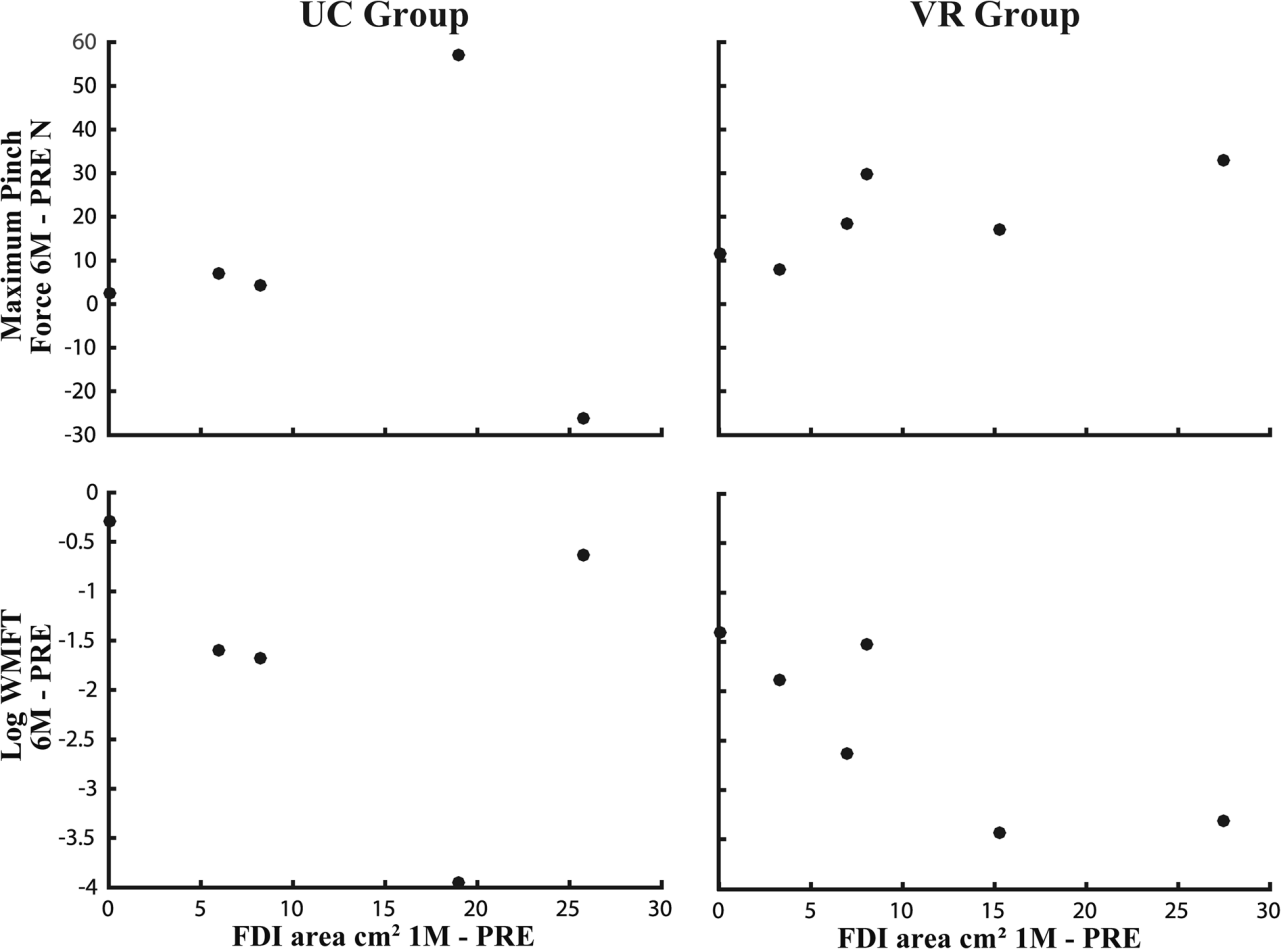
Association between change in 1M-PRE FDI area and change in 6M-PRE Pinch force and WMFT scores

The association between PRE to 1M changes in ipsilesional FDI area and PRE to 6M changes in Maximum Pinch Force and WMFT scores for both treatment groups was evaluated via scatter plots (Fig. 3). Of the four outcome measures, these two were chosen as the FDI muscle is required to pinch the index and thumb together (as measured by the Maximum Pinch Force test), and five of the fifteen WMFT items require FDI muscle usage. Statistical correlation analysis was not conducted due to the small sample sizes. PRE to 1M TMS induced map changes were chosen as we wanted to capture expansion during the enhanced period of neuroplasticity. PRE to 6M changes in outcomes were chosen as we wanted to evaluate the association between cortical reorganization during the critical, early period and long-term changes in impairment and behavior.

## Discussion

This feasibility study, initiated within 1 month post-stroke, was performed to aid in the development of a large scale RCT that we are currently conducting [(ClinicalTrials.gov (NCT03569059)]. Specifically, we compared an additional 8 h of intensive VR/robotic based upper limb training to conventional therapy. There is enhanced neuroplasticity during this early time post-stroke which is proposed to interact with training and thus lead to enhanced recovery [5]. However, there have been contradictory results from studies evaluating additional therapy provided during this time. Our approach is distinguished from previous approaches in that it provides a unique combination of focused, high intensity, and progressive training that facilitates a repeatable trajectory. Specifically, this system provides 200–300 upper extremity movements per hour of training which has been proposed to enhance neuroplasticity [18]. Based on this, we hypothesized that gains in upper limb impairment and behavior in our VR group would be greater than our UC group. We feel that the differences between the two groups in PRE to 6M change scores suggest that the hypothesis may be correct and warrants larger scale examination. Specifically, PRE to 6M change scores were significantly greater for the VR group for UEFMA scores [F(1,11) = 5.83, *p* = 0.034, η^2^ = 0.346], and Wrist AROM scores [F(1,9) = 5.342, *p* = 0.046, η^2^ = 0.372]. Notably, 6/7 of the VR subjects surpassed the MCID for the UEFMA during the training period compared to only 2/6 of the UC group. In this pilot set, improvements in PRE to 6M change scores were not siginifcantly different between groups for the WMFT, WMFT items performed, and Maximum Pinch Force. However, we were encouraged that the VR group could perform an average of 2 items more on the WMFT compared to the UC group from PRE to 6M. The ability to perform an item within 120 s at a post-test that a participant was previously unable to perform at baseline has been cited as a clinically meaningful change in persons with stroke [55].

Current evidence indicates that ipsilesional M1 excitation may be important for functional improvement of the upper limb post-stroke [56]. We thus hypothesized that enhanced long-term gains in impairment and behavior in the VR group would be associated with greater expansion in TMS based ipsilesional cortical hand representations. For the map representations, our results showed that at PRE, the cortical representation area for the FDI muscle in both groups was reduced on the ipsilesional side compared to the contralesional side. This decreased area representing the more affected hand before therapy reflects a reduced excitability of the motor cortex in the ipsilesional hemisphere that may be the result of the infarct itself [57]. Subsequently, in both groups, there was an increase in ipsilesional map size from PRE to POST, and again from POST to 1M, with a decrease thereafter. Boake et al. (2007) found a similar finding at PRE, as well as the pattern of enlargement in the ipsilesional hemisphere from PRE to POST. The reduction in area size from 1M to 6M may represent central focalization as movement stabilizes and recovery starts to plateau [57]. In contrast to our hypothesis, there were no differences between the two groups in the pattern of change for the FDI muscle representation. Statistical correlations between ipsilesional map changes and long-term changes in outcomes were not possible at this time due to small sample sizes, however associations were less variable for both the WMFT and Maximum Pinch Force scores for the VR group compared to the UC group. Larger sample sizes from the RCT will allow for a more objective evaluation of these associations.

### Study limitations

We recognize that a limitation in presenting any feasibility work is a small sample size. This precluded our ability to perform statistical correlations between TMS map changes and clinical measures. Nonetheless, this data was invaluable to develop our current RCT. As an example, for the behavioral outcome WMFT, a power analysis using these results (with an alpha of 0.05 and an estimated power of 0.8) determined that a sample size of 25 subjects would be needed in each group to show a significant difference between groups in PRE to 6M change scores. Similar analyses, as well as the effect sizes from this data, were used to determine the sample sizes for the different study arms of the current RCT, and to justify an increase in the amount of additional hours of training provided from eight to ten. Another limitation of the study was that this was a non-randomized design. However, all baseline demographic and outcome measures were statistically similar between the two groups thus eliminating potential selection bias. Additionally, although highly precise equipment was used to measure Maximum Pinch Force and Wrist AROM, a formal assessement of the reliability of our measurement technique was not conducted. Thus our method for obtaining these values could potentially have some measurement error. That being said, the same person obtained these measures throughout and followed the same set protocol at each test session to improve measurement consistency. We also plan to formally evaluate these measurement techniques during the RCT. Lastly, TMS maps for more proximal arm muscles (wrist and elbow) were not obtained with the first few subjects. This limited our ability to adequately evaluate associations between UEFMA and Wrist AROM scores and proximal TMS based muscle representations. These limitations were also addressed during the development of the RCT. Barring these limitations, the data we present here nevertheless demonstrates feasibility of conducting this intervention and multiple outcome measures (impairment, behavioral, neurophysiological) in this relatively fragile patient population, and helps guide our predictions about future results.

## Conclusions

This feasibility study initiated in the acute and early sub-acute period post-stroke compared an additional 8 h of specialized and intensive VR/robotic training to conventional rehabilitation. Long-term gains in impairment reflected by UEFMA and Wrist AROM PRE to 6M change scores was enhanced in the VR group. These greater changes in the VR group were not paralleled with augmented changes in ipsilesional FDI muscle cortical organization that were unique to this group, as similar patterns of change were demonstrated in the UC group as well. Associations between PRE to 1M change scores in ipsilesional FDI area representation and PRE to 6M change scores for the WMFT and Maximum Pinch Force measures were less variable in the VR group.

## Data Availability

The datasets used and/or analyzed during the current study are available from the corresponding author upon reasonable request.

## Abbreviations

TMS: Transcranial Magnetic Stimulation
UEFMA: Upper Extremity Fugl-Meyer Assessment
WMFT: Wolf Motor Function Test
